# Supplementary material for: On the optimization of the geometric pattern for structured illumination based X-ray phase contrast and dark-field imaging: a simulation study and its experimental validation
Source: J Synchrotron Radiat. 2026 Apr 20;33(Pt 3):806–17. doi: 10.1107/S1600577526003176 (PMC13148615; doi:10.1107/S1600577526003176)
Supplement: Supplementary file 1 [file s-33-00806-sup1.pdf]

JOURNAL OF SYNCHROTRON RADIATION

Volume 33 (2026)

Supporting information for article:

On the optimization of the geometric pattern for structured illumination based X-ray phase contrast and dark-field imaging: a simulation study and its experimental validation

Clara Magnin, Laurene Quénot, Dan Mihai Cenda, Blandine Lantz, Bertrand Faure and Emmanuel Brun

Supplementary Information : On the optimisation of the geometric pattern for structured illumination based X-ray phase contrast and dark field imaging: A simulation study and its experimental validation

Clara Magnin[a,b], Laurene Quénot[a], Dan Mihai Cenda[b], Blandine Lantz[b], Bertrand Faure[b], and Emmanuel Brun [a]

[a]Univ Grenoble Alpes, Inserm UA7, Strobe, Grenoble, France
[b]Xenocs SAS, Grenoble France

1 Comparison with explicit algorithms

To extend membrane optimization to other algorithms and not be restricted to the particular case of LCS (Magnin *et al.*, 2023), we also employed two explicit tracking algorithms: Unified Modulation Pattern Analysis (UMPA) (Zdora *et al.*, 2017) and X-ray Speckle Vector Tracking (XSVT) (Berujon & Ziegler, 2016) that might be employed to Grating Interferometry. For this comparison, the D_y displacement images of the fractal sample obtained with the spiral geometry membrane are considered. The Normalized root-mean-square error (NRMSE) index is calculated on the D_y images obtained with the 3 different algorithms according to peak-to-peak modulation distance on the membrane reference pattern. Figure 1 shows that the same trend is obtained for all of these algorithms, the optimal image quality is achieved with a modulation size ranging from 6 to 3 pixels. Among them, the LCS algorithm outperformed the others in terms of image quality. However, a detailed comparison of these different algorithms is beyond the scope of this article. For a more in-depth, quantitative comparison of these methods, we refer the reader to (Celestre *et al.*, 2024).

2 Comparison of membrane displacement in 2 or 1 directions

In this study, we focused primarily on uni-directional membrane displacements to highlight the weaknesses of different membrane geometries. The 1D motion configuration was also chosen because it is the simplest and most commonly used experimental setup for imaging devices, requiring only one motor instead of two. Figure 2 presents the image quality results obtained digitally for six different samples with four different membrane geometries, comparing bidirectional (a) and unidi-

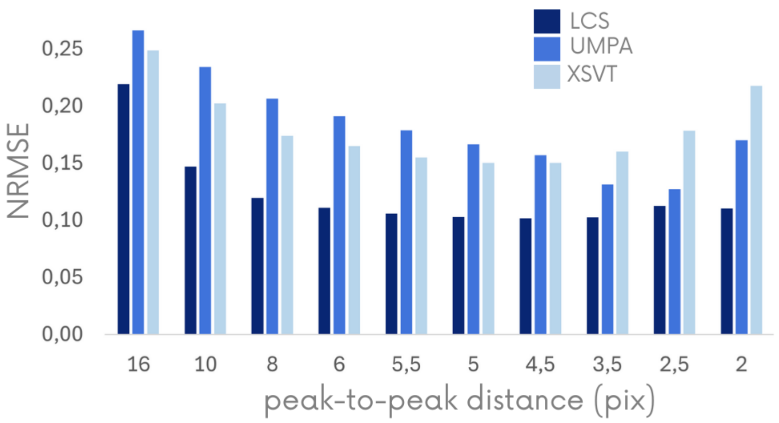

Figure 1: Normalized root-mean-square error (NRMSE) index of recovered D_y images as a function of the peak-to-peak mean distance between modulations on the membrane; comparison of 3 phase retrieval algorithms on the fractal sample with the spiral membrane. The membrane is moved in 2 directions. The lower the index, the better the image quality

rectional (b) mask displacements. The image quality was assessed using the NRMSE (Normalized Root Mean Square Error) criterion, where a lower value indicates better image quality. As shown in Figure 2, the regular membrane performed the worst in both cases (a and b), while the spiral membrane provided better image quality. The unidirectional displacement further accentuates the weaknesses of the regular membrane, leading to its exclusion from further investigation due to the poor quality of the images it produced.

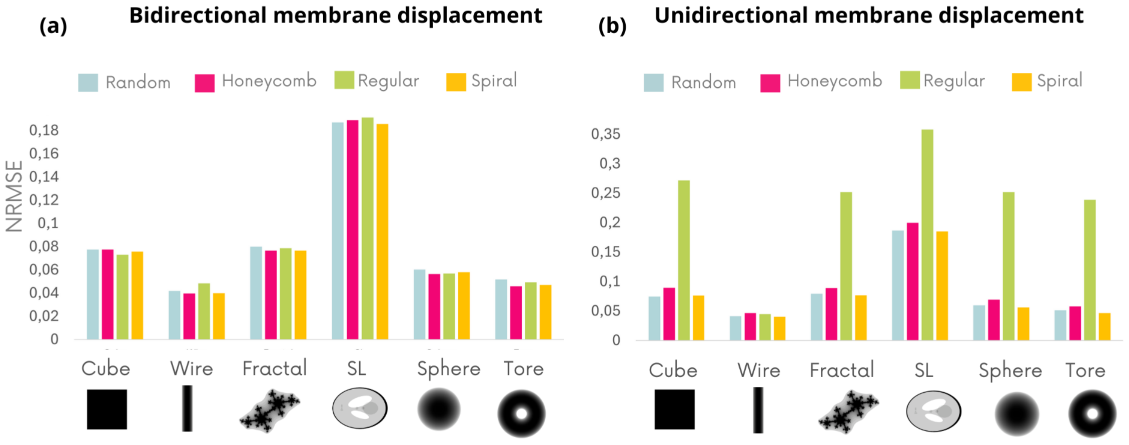

Figure 2: NRMSE index calculated on the 6 samples D_y images retrieved according to different membranes geometries. The membrane is moved in 2 directions (a) and one direction only (b). The lower the index, the better the image quality

3 Comparison of spiral geometries

Generally speaking, a spiral is a curve that wraps around a fixed point while moving progressively away from it. Mathematically, a spiral can be described in a polar coordinate system by an equation of the form :

$$r = f(\theta)$$

with r the distance from the point to the center, θ the angle in radians, and $f(\theta)$ the function that defines how r varies with angle. Several spiral geometries exist, and in this study two spiral patterns were considered : the Vogel and Archimedes spirals.

- The Vogel spiral equation

$$c^2\varphi^2\theta = 2\pi\rho^2$$

  with ϕ the golden number, is established by Helmut Vogel (Vogel, 1979) and is a special case of the Fermat spirals of equation $\rho^2 = a^2\theta$ This motif reproduces the patterns of sunflowers, whose spatial arrangement is known to optimize light capture by the heart of the flower.

- The Archimedean spiral is a spiral that winds uniformly, at a constant pitch, around a central point. It is defined by the equation

$$r = a + b\theta$$

  where a and b are constants. In this spiral, the distance between successive turns is constant.

Figure 3 shows all the membrane topologies used, detailing the different spiral membrane patterns in sub-Figure (d,e,f).

The Vogel spiral was the first to be implemented in this study because it is known to maximize space paving and is found in nature, particularly in the geometry of sunflower seed heads. This first spiral was therefore implemented numerically and used throughout the simulation-based portion of the study. The Archimedean spirals were later used to compare the performance of the different membrane geometries by simulation and experiment.

A comparison of the image quality achieved with the different membrane patterns was conducted through simulation to ensure that the different spiral membranes resulted in similar image quality. Figure 4 shows the D_y images obtained numerically on the fractal object using the three membrane geometries: 2 Vogel spirals (b,c), with slightly different parameters for the space between modulations, and Archimedes spiral (d). Visually, the noise and resolution of the images appear very similar. This observation is confirmed by the FRC values achieved by the images (e), which are comparable (on average 0.43 ± 0.04 pix^{-1}) and, in all cases, outperform the values obtained previously with the random and honeycomb membranes (0.31 and 0.37 pix^{-1}) presented in Figure 5 of the article.

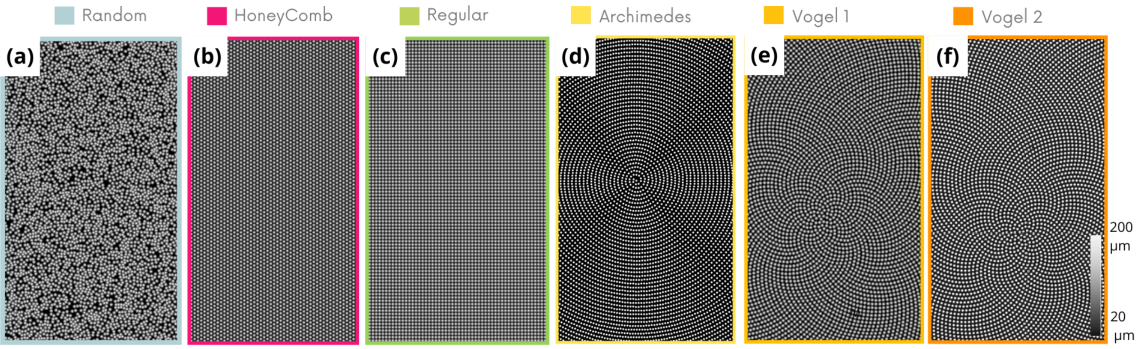

Figure 3: Membrane topologies used in this study, (b, d, e) were machined for experimental use.

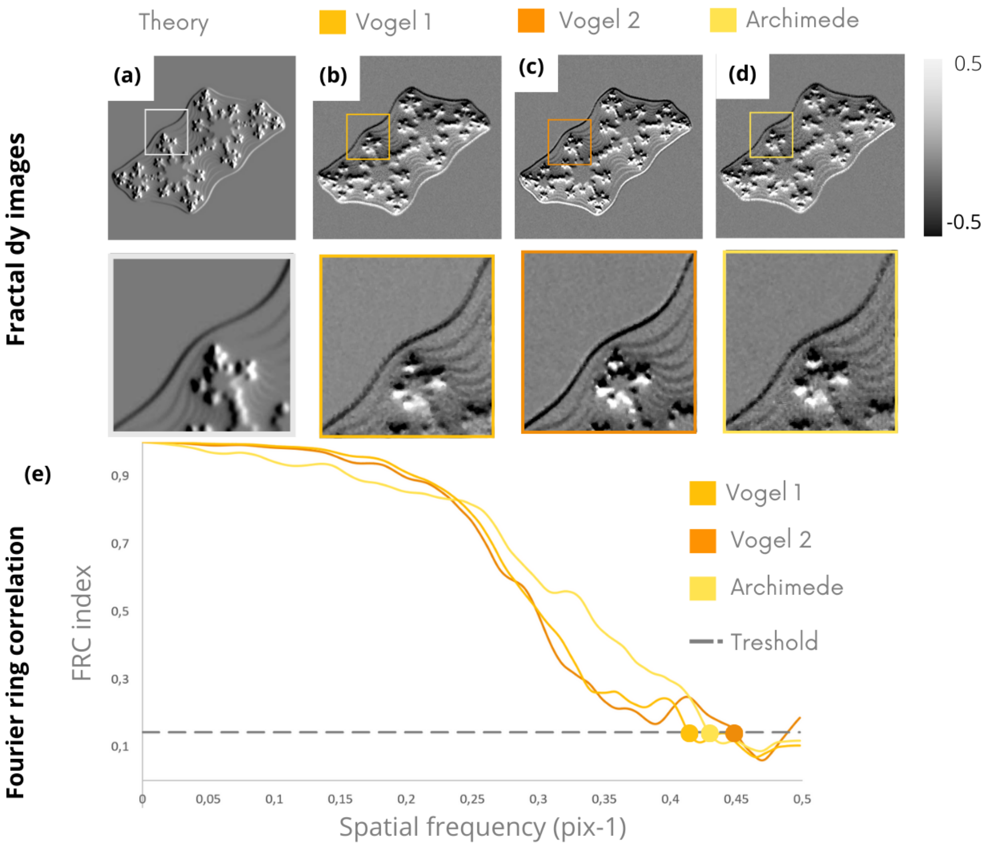

Figure 4: D_y images comparison on the fractal sample obtained in theory (a) and with the different spiral membrane geometries (b,c,d). FRC index computed on the 3 D_y images (e) with a threshold at 1/7

References

Berujon, S. & Ziegler, E. (2016). *Physical Review Applied*, **5**(4), 044014.

Celestre, R., Quenot, L., Ninham, C., Brun, E. & Fardin, L. (2024). *arXiv preprint arXiv:2404.11633*.

Magnin, C., Quénot, L., Bohic, S., Mihai Cenda, D., Fernández Martínez, M., Lantz, B., Faure, B. & Brun, E. (2023). *Optics Letters*, **48**(22), 5839–5842.

Vogel, H. (1979). *Mathematical Biosciences*, **44**(3), 179–189. https://www.sciencedirect.com/science/article/pii/0025556479900804

Zdora, M.-C., Thibault, P., Zhou, T., Koch, F. J., Romell, J., Sala, S., Last, A., Rau, C. & Zanette, I. (2017). *Physical review letters*, **118**(20), 203903.
